# The Natural History and Treatment Options for Unruptured Intracranial Aneurysms

**DOI:** 10.1155/2012/898052

**Published:** 2012-02-28

**Authors:** Joshua E. Loewenstein, Shaneze C. Gayle, E. Jesus Duffis, Charles J. Prestigiacomo, Chirag D. Gandhi

**Affiliations:** ^1^Department of Neurological Surgery, New Jersey Medical School, University of Medicine and Dentistry of New Jersey, Newark, NJ 07101, USA; ^2^Department of Radiology, New Jersey Medical School, University of Medicine and Dentistry of New Jersey, Newark, NJ 07101, USA; ^3^Department of Neurology & Neuroscience, New Jersey Medical School, University of Medicine and Dentistry of New Jersey, Newark, NJ 07101, USA

## Abstract

Recent advances in angiographic technique have raised our awareness of the presence of unruptured intracranial aneurysms (UIAs). However, the appropriate management for these lesions remains controversial. To optimize patient outcomes, the physician must weigh aneurysmal rupture risk associated with observation against the complication risks associated with intervention. In the case that treatment is chosen, the two available options are surgical clipping and endovascular coiling. Our paper summarizes the current body of literature in regards to the natural history of UIAs, the evolution of the lesion if it progresses uninterrupted, as well as the safety and efficacy of both treatment options. The risks and benefits of treatment and conservative management need to be evaluated on an individual basis and are greatly effected by both patient-specific and aneurysm-specific factors, which are presented in this paper. Ultimately, this body of data has led to multiple sets of treatment guidelines, which we have summated and presented in this paper.

## 1. Introduction

Despite an abundance of published data, management of patients with unruptured intracranial aneurysms (UIAs) remains controversial. Advances in imaging and more frequent use of CTA and MRA over the last two decades have elucidated the pervasiveness of intracranial aneurysms. Furthermore, these imaging techniques have been refined over the years giving them greater sensitivity and specificity and increasing the number of cerebral aneurysms found incidentally. Ultimately, physicians have realized that UIAs are rather common. Imaging studies have reported frequencies of 0.5% to 2%, while autopsy studies have reported frequencies of 1% to 9% [1].

The inherent threat of UIAs is rupture and subsequent subarachnoid hemorrhage (SAH). Recent studies of aneurysmal SAH, which accounts for around 80% of nontraumatic SAH, have reported 1-month case mortality rates as high as 30% to 50% [2–4]; these devastating outcomes are still extraordinary. Poor neurologic and functional outcomes among patients whom have suffered SAH are due to initial hemorrhage, early rebleeding, and delayed cerebral ischemia resulting from cerebral vasospasm, microvascular dysfunction, and complex neuronal-glial interactions [5]. Therefore, preventive measures must be taken to better predict rupture risk of UIAs.

Universal treatment protocols for patients with UIAs have yet to be established. Intervention by surgical clipping or endovascular coiling constitutes one strategy, while others have opted for conservative management and observation. Chief in this clinical evaluation is balancing the risk of treatment with that of observation. A variety of largely nonrandomized studies have implicated different patient characteristics (age, medical condition, history of SAH, etc.), aneurysm characteristics (size, location, morphology, neck size, etc.) and management factors (surgical team, treating hospital), as being influential in the natural history and outcomes following treatment [6]. This paper will thoroughly review the current literature regarding the natural history and treatment options for UIAs and present current guidelines for treatment.

## 2. Natural History of UIAs

Characterizing the aneurysmal rupture risk of UIAs, known as their natural history, is a task that has proven very difficult over the years. While much has been revealed about UIAs from retrospective review, inconsistencies and methodological flaws in studies regarding UIAs have resulted in a poorly defined natural history. Several reasons explain why this uncertainty exists. Paramount among them is the fact that natural history studies of UIAs only evaluate those patients for whom conservative management was decided. It has therefore been argued that data from these studies do not represent the behavior of all UIAs, only certain types. The current body of published literature has thoroughly evaluated aneurysms on the basis of many different risk factors, which can be roughly divided into patient-specific and aneurysm-specific factors.  Table 1 lists the important factors affecting the natural history of UIAs and provides key findings related to each.

### 2.1. Size as a Risk Factor for Rupture

In almost all natural history studies to date, size has been found to be a significant predictor of aneurysm rupture. In general, the notion that large UIAs have a greater rupture risk than small UIAs is widely accepted. However, an exact size threshold above which UIAs impart significantly greater rupture risk has yet to established [21].

The largest multicenter study of UIAs at the time, the ISUIA (International Study of Unruptured Intracranial Aneurysms), aimed to characterize the natural history of unruptured aneurysms. The retrospective cohort of the ISUIA study found that, in patients without previous history of SAH, UIAs of less than 10 mm in diameter had a rupture rate (RR) of 0.05% per year. It also found that UIAs more than 10 mm in diameter had an RR of less than 1% per year. The authors initially concluded that UIAs were more benign than previously reported [7]. The follow-up, prospective arm of the ISUIA performed a similar analysis on those patients who were chosen for conservative management but employed different size thresholds of 7 mm, 12 mm, and 25 mm. Of the 1077 conservatively managed patients who displayed no prior history of SAH, 41 suffered aneurysm rupture of which only 2 were from aneurysms less than 7 mm [8]. After analyzing both branches of their study, the investigators of the ISUIA concluded that size certainly plays an essential role in the assessment of rupture risk, but cannot be evaluated independent of all other factors.

More recent natural history studies continue to reinforce the importance of size in evaluating rupture risk. A study by Ishibashi et al. classified aneurysms by size, small (<5 mm), medium (5–9.9 mm), large (>10 mm), and giant (>25 mm), and found annual RRs of 0.8%, 1.2%, 7.1%, and 43.1%, respectively [9]. Additionally, a meta-analysis performed by Wermer et al. of more than 4700 patients and 6500 UIAs taken from 19 studies worldwide revealed that UIAs from 5 mm–10 mm and >10 mm had rupture risks 2.8 times and 5.2 times greater than aneurysms <5 mm. While it is apparent that aneurysmal size is significant, the inconsistent statistical analyses among these studies make defining a critical size threshold for UIAs difficult.

### 2.2. Enlargement as a Risk Factor for Rupture

The growth of UIAs over time is important to the clinician for multiple reasons. For one, it has been hypothesized that an increase in UIA size might be indicative of forthcoming rupture, even in smaller aneurysms. One study by Yasui et al. specifically analyzed 25 cases of SAH caused by rupture of a conservatively managed intracranial aneurysm. The results of this study found that in most cases the diameter at time of rupture was greater than the diameter at initial diagnosis. Furthermore, of these 25 cases, 16 dealt with aneurysms that were <5 mm at initial diagnosis [10]. Therefore, despite initial size, it is important to understand aneurysmal growth patterns to guide maintenance and followup.

Another study, completed in 2008 by Burns et al., evaluated enlargement as a risk factor for aneurysm rupture. The study retrospectively identified 165 patients with 191 UIAs followed with serial MR angiography and discovered that, after a median follow-up period of 47 months, 20 aneurysms (~10%) grew. Frequency of enlargement was 6.9%, 25%, and 83% for aneurysms <8 mm, 8 to 12 mm, and ≥13 mm, respectively [1]. Of the variables evaluated, which included patient age and aneurysm location, original aneurysm diameter was the only independent predictor of enlargement. Similarly, a study by Matsubara et al. focused on the risk factors for UIA growth when the chosen angiographic technique was CTA. Growth was identified in 6.4% of all aneurysms, with aneurysms of 2–4 mm, 5–9 mm, and 10–20 mm, producing growth frequencies of 2.4%, 9.1%, and 50%, respectively [11]. It can thus be concluded that having larger aneurysms will increase the risk of enlargement and consequently might accelerate aneurysmal rupture.

### 2.3. Previous Subarachnoid Hemorrhage as a Risk Factor for Rupture

Among the chief goals of the ISUIA was better understanding the role that previous medical history, particular one with aneurysmal SAH, had on future rupture risk. Consequently, the retrospective component of the ISUIA, stratified its patients into two groups, with group 1 consisting of patients who had no history of SAH and group 2 consisting of patients who had a history of SAH from a different aneurysm. The patients in group 2 with UIAs <10 mm possessed an RR of 0.5% per year, a figure which is ~11 times higher than that for patients in group 1 with aneurysms of the same size [7]. The same groupings were made in the prospective branch of the ISUIA to allow for similar analysis, but different size thresholds were employed. This branch of the study reported that patients in group 2 with aneurysms <7 mm had similar RR as patients in group 1 with aneurysms >7 mm, both of which were significantly higher RRs than patients in group 1 with aneurysms <7 mm [8]. Overall, the natural history data from both branches of the ISUIA confirmed hypotheses, which asserted that a prior history of SAH increases aneurysmal rupture risk.

### 2.4. Location as a Risk Factor for Rupture

As the various cerebral blood vessels have different characteristics and experience different hemodynamic conditions, one would expect location of the aneurysm to influence its natural history. Some studies had originally identified aneurysms at the anterior communicating and pericallosal arteries as being at high risk of rupture [12, 22]. However, the cerebral arteries that are now considered most hazardous are located in the posterior circulation and include the tip of the basilar artery, posterior cerebral artery, vertebrobasilar distribution, and the origin of the posterior communicating artery [23]. In fact, the only clear, independent predictor of future rupture among patients with a history of SAH in the ISUIA was basilar tip location. A study performed by Ishibashi et al. found that posterior circulation location was an independent predictor of aneurysm rupture and that aneurysms in these locations had a hazard ratio of 2.9 [9]. Likewise, the meta-analysis performed by Wermer et al. in 2007 found posterior circulation location to be a statistically significant risk factor for aneurysmal rupture [13].

On the other hand, UIAs in the intracavernous ICA have been found to be more benign. This portion of the ICA is seldom located in the subarachnoid space, and thus rupture of these aneurysms rarely causes SAH [14]. Further, their natural history suggests that a large number of such aneurysms will remain clinically asymptomatic [24] and carry significantly lower rates of rupture and mortality compared to aneurysms situated in the subarachnoid compartment [25]. The prospective branch of the ISUIA found that intracavernous aneurysms had a 0.15 relative risk compared to aneurysms in other portions of the ICA [8].

### 2.5. Morphological Characteristics as Risk Factors for Rupture

Atypical aneurysm morphology, such as multilobulation and loculations, daughter sac formation, and other unique hemodynamic factors play roles in raising the risk of rupture [26]. Hademenos et al. completed a study involving 74 patients, with 40 ruptured and 34 unruptured aneurysms evaluating location and lobulations [15]. They reported that 16 (84%) of 19 multilobulated aneurysms had ruptured, compared with only 24 (44%) of 55 unilobular aneurysms. Posterior circulation aneurysms were also noted to be of higher risk with multilobular posterior circulation aneurysms at the highest risk of all [27]. Similarly, Beck et al. explored the difference between ruptured and unruptured aneurysms in regards to lobulation, the presence of a daughter sac, or the shape as measured by the height/neck ratio [16]. They used biplane digital subtraction angiography (DSA) to analyze the aneurysms in their retrospectively analysis of 124 patients including 53 unruptured and 94 ruptured aneurysms. 10% of unruptured aneurysms showed a multilobular appearance on DSA compared with 20% of ruptured aneurysms. In the 5–9 mm aneurysm group, multiple lobes were found in 26% of ruptured aneurysms. A height/neck ratio of less than 1.5 was not found in any of the unruptured aneurysms but was found in 21% (12/57) of the ruptured aneurysms. The data supported the theory that aneurysms with multilobular appearance have greater frequency of rupture.

Given that intracranial aneurysms are more commonly found at either bi- or trifurcations or at regions of high impact from flowing blood, the relationship of aneurysm to the surrounding vasculature has been examined [27]. With respect to the surrounding vasculature, Sadatomo et al. reported on the relationship among aneurysm neck, parent artery, and daughter branches in 44 cases (20 unruptured, 24 ruptured) of middle cerebral artery aneurysm [28]. When the neck was located on the extension of the midline of the parent artery, it was defined as Type C (19 cases); when it was not, it was defined as Type D (25 cases). They found that in all cases, Type D aneurysms were located on the side of the daughter artery with a narrower angle to the parent artery. In >90% of the cases, the aneurysm was located on the side of the smaller artery, suggesting that the dominant artery provided the hemodynamic force for aneurysm formation and likely increased rupture risk. Furthermore, Type C as well as a high dome/neck ratio is associated with increased aneurysmal rupture risk [28].

Novel technology providing three-dimensional angiographic images has facilitated visualization of cerebral vasculature and helped investigators analyze aneurysm morphology. Dhar et al. utilized this technology to evaluate UIAs based on eight parameters, three of which incorporate parent vessel geometry and had not been previously explored (vessel angle, aneurysm (inclination) angle, and (aneurysm-to-vessel) size ratio) [17]. Of the parameters examined, size ratio and aneurysm angle (with respect to the parent artery) had the strongest correlation to rupture risk. Still, another group of investigators found that the undulation index and Nonsphericity index, different metrics of aneurysm morphology, have strong correlations to UIA rupture risk [18]. Nonsphericity index characterizes the deviation of UIA from that of a perfect sphere by using aneurysm volume and surface area, and undulation index characterizes the number and size of concave regions on the surface of the aneurysm.

### 2.6. Patient-Specific Characteristics as Risk Factors for Rupture

Patient factors such as age, sex (females are 1.5–3 times more likely to have a ruptured aneurysm), and comorbidities, such as hypertension and cigarette smoking, influence whether an aneurysm will rupture [19]. 87 patients were involved in a long-term study conducted by Juvela et al.. 79 of the patients had ruptured aneurysms clipped at the start of followup. There were 111 unruptured aneurysms as well as an additional 7 patients (2 with and 5 without unruptured aneurysms) who developed new aneurysms. The patients were followed from the 1950s to the 1970s until death, subarachnoid hemorrhage, or until the last contact; the mean follow-up period was 18.9 years. Unruptured aneurysms increased in size ≥1 mm in 39 of the 87 patients (45%) and ≥3 mm in 31 (36%). New aneurysms were found in 15 of the 89 patients and in 5 without an unruptured aneurysm at the beginning of followup. After adjustment of age, the significant risk factors for increased risk of intracranial aneurysm formation and growth were cigarette smoking and female sex [20]. Cessation of smoking should therefore be employed for all patients with unruptured aneurysms and possibly also for those with a prior subarachnoid hemorrhage.

## 3. Treatment Options for UIAs

 The most appropriate treatment option for any UIA is that which provides an optimal balance of procedural safety and long-term efficacy based on patient and aneurysm characteristics. Currently, there are two available options for treating UIAs, microsurgical clipping and endovascular coiling. For each of these treatment modalities, recurrence, retreatment, and percent occlusion data have been gathered to quantify efficacy, while morbidity/mortality and procedural complication rates have been utilized to quantify safety. Although the merits of each treatment are well documented, the superiority of one treatment over the other remains controversial. This section of the paper presents both the current data regarding each treatment and the proposed guidelines set forth to help physicians optimize patient outcomes.

### 3.1. Surgical Treatment

Traditionally, surgical clipping has been viewed as being highly efficacious, but carrying greater risk due to the neurological complications associated with open neurosurgery. Efficacy of this treatment is illustrated by a study performed between 1998 and 2001 that explored the need for cerebral angiography following surgery for saccular aneurysms. Of the 315 surgically clipped UIAs in this study, 287 were completely occluded, a 91% complete occlusion rate [29]. On the other hand, safety concerns associated with surgical treatment are shown in a report analyzing data from twenty-one single-center and eight multicenter studies of surgical clipping from 1991 to 2003 [30]. In this study, mortality rates ranged from 0% to 6.9%. The same study found the adverse outcome rate (AOR) to range between 0% and 25.1% with a cumulative AOR of 17.8%. The inconsistency in this analysis might also represent the varying levels of expertise among neurosurgical teams at different institutions.

Another goal of the prospective branch of the ISUIA was to obtain a more comprehensive assessment of the surgical risks of aneurysm clipping. Among the 1917 patients who underwent surgical clipping, the study found a 1-year mortality rate of 2.3% and a 1-year morbidity rate of 12.1%. The study also found that increasing aneurysmal size and patient age as well as location in the posterior circulation (particularly basilar tip and posterior communication artery) are indicators of poor outcome following surgical treatment [8]. Consequently, older patients with large, posterior circulation aneurysms are most likely better candidates for endovascular treatment.

Based on the natural history data presented in the ISUIA, Krisht et al. calculated that (according to size parameters) patients on their service whom had chosen surgical treatment would have had a 10-year cumulative bleeding-related mortality and severe morbidity rate of no less than 7.5% had they chosen conservative management. This figure is significantly greater than the 4.2% combined morbidity and mortality rates that their patient population actually experienced, suggesting that surgical treatment may represent a superior approach to conservative management in patients with life expectancies greater than 10 years [31].

 More recent studies are showing that refinement of microsurgical technique is leading to safer, more efficacious treatment of UIAs. One study followed a series of 450 aneurysms treated with surgical clipping by one neurosurgeon immediately upon completion of neurosurgery training. With 6-month morbidity and mortality rates of 1.06% and 0.27%, respectively, it is clear that, despite an increase in endovascular procedures, given proper mentorship and resources prevailing neurosurgeons can achieve acceptable results when treating UIAs surgically [32]. Even in populations widely recognized as better candidates for endovascular treatment, such as those over 65 years old, low morbidity rates have legitimized surgical clipping as a safe option [33].

 As previously mentioned, the expertise and experience of the neurosurgical team and treating hospital will also affect postoperative outcomes. A study at the Cleveland Clinic of 499 aneurysms, treated by 10 neurosurgeons, found that the number of aneurysms clipped by a specific neurosurgeon was a strong predictor of positive outcome, along with patient age and aneurysm size [34]. In the state of New York, hospitals that performed greater than 30 craniotomies per year saw 43% reductions of in-hospital mortality for the treatment of both ruptured and unruptured aneurysms [35]. Similarly, a study of the national inpatient sample (NIS) from 1996 to 2000 showed that patients treated in high-volume hospitals (treated ≥20 UIAs per year) rather than low-volume hospitals (treated ≤4 UIAs per year) had lower adverse outcomes and lower mortality rate (1.6% versus 2.2%) [36]. Consequently, it is increasingly important that neurosurgeons understand the limitations of both their technical capability and their available resources when making treatment decisions regarding UIAs.

### 3.2. Endovascular Treatment

**Figure 1:**
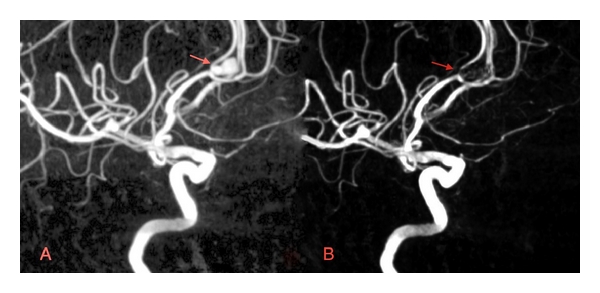
(a) Preoperative 3D digital subtraction angiogram (DSA) of a left 6.6 mm pericallosal UIA. 54-year-old female patient had prior history of SAH and harbored multiple smaller aneurysms on the right. Endovascular coiling was chosen. (b) Postoperative 3D DSA. Treatment achieved 99% occlusion.

**Figure 2:**
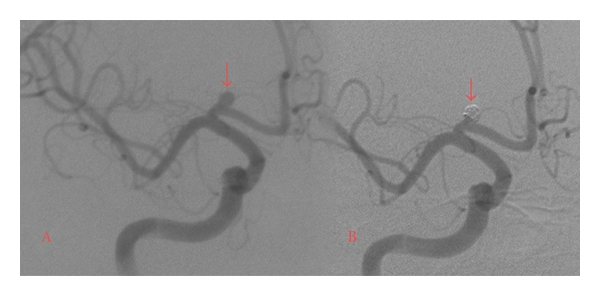
Preoperative DSA of a 4.1 mm × 3.2 mm ACOM aneurysm. This specific UIA was chosen for endovascular treatment due to enlargement and visual field deficits. (b) Postoperative DSA. Endovascular coiling produced near complete occlusion.

Since its conception and implementation about two decades ago [37, 38], endovascular coil embolization has become a primary treatment in the management of UIAs. In many institutions worldwide, endovascular coiling has become the preferred treatment for UIAs for which intervention is indicated [39]. Figures 1 and 2 provide examples of UIAs successfully treated with endovascular coil embolization. Whereas physicians often worry about the safety of open neurosurgery, the main concern with endovascular techniques is efficacy. Early efficacy reports perpetuated this viewpoint. A meta-analysis including 46 studies of patients treated with endovascular coiling from 1990 to 1997, revealed a rather low complete-obliteration rate of 54% [40]. Similarly, the prospective arm of the ISUIA reported a 55% complete obliteration rate, a 24% incomplete obliteration rate, and no obliteration in 18% of the 451 patients who were treated endovascularly [8].

In addition to degree of occlusion, the efficacy of treatments for UIAs, especially endovascular coiling, has been measured by recanalization and retreatment rates. These rates quantify the long-term effectiveness of coiling and help surgeons and physicians understand how much treatment is altering the natural history of these lesions. One multicenter study published by Gallas et al. in 2009 analyzed intracranial aneurysms treated within five-year period from 1998 to 2003 and included a mean follow-up time of 55.6 months [41]. Of 232 UIAs treated by endovascular coiling in this study, 172 were completely occluded and, of the rest, 18 (7.8%) required retreatment, most during the first postoperative year. These complete occlusion and retreatment rates are certainly higher than those for patients who underwent surgical clipping.

 To confirm the notion that endovascular procedures provide a safe alternative for treating UIAs, many studies have investigated the morbidity and mortality rates of patients who underwent endovascular coiling. As part of the prospective branch of the ISUIA, 451 patients were chosen to undergo endovascular coiling. Among these patients, there was a 1-year surgery-related mortality rate of 3.1% and a 1-year morbidity rate of 9.5% [8]. A year later, Lanterna et al. analyzed thirty studies of endovascular coiling, published between 1990 and 2002, and revealed case-fatality and permanent morbidity rates of 0.6% and 7.0%, respectively. To appreciate the effect of advancing technology and technical refinement, the investigators divided the publications by midyear of the study and found that studies before 1995 reported a morbidity rate of 8.6%, while the morbidity rate of studies after 1995 had dropped to 4.5% [42]. Another more recent meta-analysis performed by Naggara et al. looked at similar studies performed in 17 different countries from 2003 to 2008 and found unfavorable outcomes occurred at a rate of 4.8% [43]. Ultimately, these data do seem to indicate that endovascular coiling provides a safe alternative to coiling by imparting lower complication rates upon patients.

 While endovascular treatment of UIAs is now widely used, certain aneurysmal morphologies and anatomical features, particularly a wide neck, render some aneurysms technically difficult to treat endovascularly. To facilitate endovascular coiling of aneurysms with broad necks, Moret et al. extended a previously utilized temporary balloon-inflation technique to the treatment of UIAs and named it balloon remodeling [44]. One part of the ATENA (Analysis of Treatment by Endovascular Approach of Nonruptured Aneurysms) Study explored the safety of the balloon remodeling technique. The morbidity and mortality rates of UIAs treated with balloon remodeling were 2.3% and 1.4%, respectively, compared to 2.2% and 0.9% for those receiving standard endovascular coiling, a difference which was not statistically significant [45]. In terms of efficacy, a review comparing aneurysms treated with balloon remodeling versus standard endovascular treatment, in studies published from 1997 to 2006, revealed significantly higher initial and follow-up aneurysm occlusion rates for those aneurysms treated with balloon remodeling [46].

**Figure 3:**
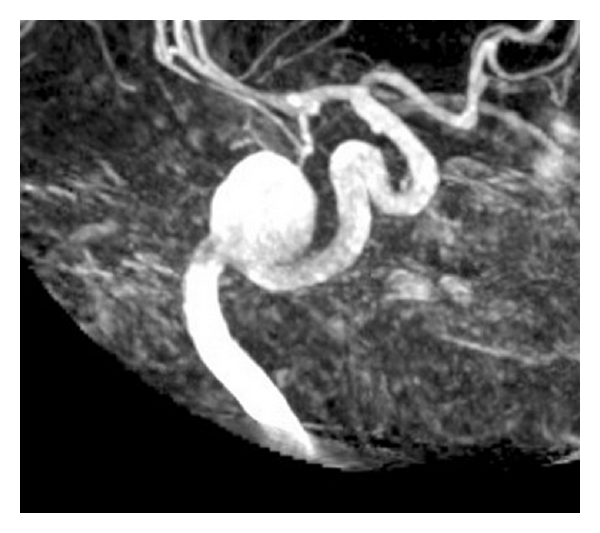
3D DSA of a giant, left, petrous ICA aneurysm. Stent-assisted coiling was performed on this patient.

 Another adjunctive therapy for wide-neck UIAs is microcatheter-delivered stenting. The hope among neurosurgeons is that stent-assisted coil embolization (SAC) may improve long-term durability and effectiveness by minimizing herniation and increasing packing density [47]. After establishing procedural safety and periprocedural effectiveness [48], Sedat et al. reported that long-term complete aneurysmal occlusion occurred in 71% of patients, with aneurysmal regrowth in 4 out of 38 patients at first angiographic followup and no regrowth in any other followups [49]. Still, other investigators wanted to fully value the safety and efficacy of SAC and subsequently created the Interstate Collaboration of Stent Coiling (ICES). Initial results of the ICES study concluded that this technique was helpful for treatment of UIAs but not RIAs and produced morbidity and mortality rates of 2.8% and 2.0%, respectively [50]. The midterm report of the ICES study, which then encompassed 216 SAC-treated aneurysms, reported that 40% of aneurysms demonstrated complete occlusion and 88% had ≥90% aneurysm occlusion, illustrating that SAC is viable option for wide-neck aneurysms [51].  Figure 3 presents a UIA for which SAC was the chosen treatment modality.

### 3.3. Comparative Studies of UIA Treatment

For many years, microsurgical clipping of aneurysms was considered the “gold standard” of treatment for UIAs. However, emerging attitudes favoring noninvasive procedures have coincided with a steep increase in gross number and variety of aneurysms treated endovascularly [52]. According to NIS records, the fraction of UIAs treated with endovascular coiling has risen from 20% in 2001 to 63% in 2008 [53]. As shown above, the amassed literature comparing the two treatment options, composed primarily of nonrandomized, retrospective studies, varies considerably in regard to safety and efficacy data. Consequently, uncertainty over the superiority of one method over another remains, and approaches to treatment are often based largely upon opinion or personal experience rather than scientific evidence.

In order to settle this controversy, many studies have directly compared endovascular coiling to surgical clipping. A multicenter retrospective cohort study of over 2500 UIAs treated with either coiling or clipping between 1998 and 2000 revealed significantly lower morbidity (6.6% versus 13.2%) and mortality (0.9% versus 2.5%) in those patients treated endovascularly [54]. Furthermore, a recent analysis of the NIS discovered that the percentage of patients discharged to long-term care after surgical clipping was 14.0%, compared to 4.9% for those treated with coiling, and also found an overall decrease in adverse outcomes in patients treated for a UIA from 14.8% in 2001 to 7.6% in 2008. They attributed the increasing safety of these types of procedures to advances in endovascular technique and expertise [52]. Another publication from the same study of the NIS stratified patients by age and revealed that the higher morbidity and mortality rates of surgically clipped patients were more pronounced in the elderly population [55]. This demonstrated safety of coiling relative to clipping might explain its increased use over the past decade.

While numerous RCTs have investigated the treatment safety and efficacy of RIAs [56–58], substantial obstacles have prevented completion of RCTs comparing the treatment options of UIAs. Initiated in 2006, the TEAM trial aimed to be the first true, international RCT comparing endovascular coiling to conservative management [59]. However, in 2009, the study was terminated due to poor subject recruitment. Among many other reasons, the authors cite a pervasive distrust between scientific research and clinical care as an explanation for the trials failure [60]. Another, seemingly more practical, RCT called the CURES (Canadian Unruptured Endovascular versus Surgery) trial is expected to evaluate which intervention, coiling or clipping, leads to better morbidity, mortality, and clinical efficacy in patients chosen for intervention. Currently the investigators are still in the pilot phase of the trial in which they will assess the study's feasibility and determine the incidence of treatment failure, defined as incomplete aneurysm obliteration, major saccular aneurysm remnant, or SAH within a year of treatment [61].

The clinical evaluation of UIA patients also must consider the economic burdens of each treatment. This reality is particularly valid when the treatment rationale is unclear, as is the case in UIA treatment. Investigators in Europe initially reported that there was no significant difference between cost of endovascular coiling and surgical clipping [62]. However, to explore costs in the United States healthcare system, an analysis of the NIS published in 2011 explored the differences in regards to length of hospitalization and hospital charges between patients who underwent clipping versus coiling. It found that UIAs patients treated with endovascular coiling had shorter lengths of stay and, on average, $11,263 less total hospital charges compared to patients treated with surgical clipping [63].

### 3.4. Future Directions of UIA Treatment

The endovascular approach to UIA treatment, having only been FDA approved since the early 1990s, remains a relatively new option. As such, there is still a great deal of research and innovation occurring in the field, which frequently leads to new technologies. The hope with these advances is that neurosurgeons will be able to treat a wider spectrum of intracranial aneurysms with greater safety and efficacy, lowering incomplete occlusion and recanalization rates while maintaining acceptable levels of morbidity and mortality.

While primarily used in the treatment of arteriovenous malformations (AVMs), liquid embolic systems such as Onyx HD-500 (EV3, Irvine, CA, USA) are becoming an increasingly popular option in the treatment of wide-necked intracranial aneurysms. Compared with standard platinum coil embolization, liquid embolic systems are posited to provide greater neck and parent vessel reconstruction while also inducing greater neoendothelization of the aneurysm neck. One retrospective study of 84 wide-necked intracranial aneurysms treated with Onyx HD-500 (74 of which were unruptured) found there to be an overall complete occlusion rate of 65.5% immediately following the procedure. Furthermore, 68.2% of the incompletely occluded aneurysms progressed to complete occlusion by 6 months, and, by 18 months, 90.3% of all aneurysms achieved complete occlusion. Procedural morbidity and mortality rates in this study were 7.2% and 2.9%, respectively [64]. A more recent single-center, prospective study reported that all 13 of their Onyx HD-500 treated, wide-necked UIAs achieved greater than 90% occlusion immediately postoperation. This level of occlusion was maintained in all patients at 6-month followup [65].

Another, more recently approved, endovascular approach is parent vessel reconstruction using a pipeline (EV3, Irvine, CA, USA) embolization device (PED). Similar to the microstent, the PED is a self-expanding, microcatheter-delivered, cylindrical mesh device. However, its 30–35% metal surface area coverage (compared to the 6–9% coverage of self-expanding microstents) allows it to function as a stand-alone reconstruction device, diverting blood flow away from the aneurysm [66]. In its early stages of development, the PED has been utilized in cases when standard coil embolization has been largely ineffective, such as wide-necked, large or giant, and nonsaccular, fusiform aneurysms. In a recent study of 53 patients harboring 63 aneurysms approved for compassionate use of PED treatment for large or giant, wide-necked, nonsaccular aneurysms, no major complications were noted. Complete occlusion occurred in 56% of aneurysms at 3-month followup, 93% at 6-month followup, and 95% at 12-month followup [67]. One major concern with PED treatment is delayed occlusion, especially since, at present, it is primarily being utilized to treat high-rupture-risk aneurysms. Still, the results of this study indicate that PED could become a safe and effective treatment of more complex UIAs.

### 3.5. Treatment Recommendations

 In 2000 the American Heart Association's Stroke Council published a list of recommendations to provide the physician with a framework for making appropriate treatment decisions. In these recommendations, the council emphasizes patient age in the clinical evaluation, noting that the higher treatment risks and shorter life expectancies of older people favor observation, especially in the case of small, asymptomatic UIAs. For example, conservative management might be suggested for an older patient with a UIA ≥10 mm, while treatment might be recommended for young patients with UIAs between 5 mm and 9 mm. Furthermore, the council recommends treatment for all patients with prior history of aneurysmal SAH as well as all symptomatic, intradural UIAs and UIAs ≥10 mm, unless age or medical condition limit treatment benefits. Additionally, special consideration should be given to aneurysms with unusual morphology such as multilobulations as well patients with a family history of aneurysms or SAH [68].

However, the wealth of recently published data on UIA treatment has led many physicians to implement their own protocols based on the studies they find most credible among the literature. Many emphasize aneurysm size in their clinical evaluation, but these decisions are complex and require consideration of many other factors as well. The first decision is whether to treat. At Columbia University as of 2007, the neurosurgical team reported that their interpretation of the literature and the experience of their microsurgeons and endovascular surgeons have led them to believe that all symptomatic aneurysms, aneurysms from 5–10 mm in patients under 60 years old, and aneurysms >10 mm in patient under 70 years old should be strongly considered for treatment [69].

Comparatively, the management summary submitted by Robert Brown, a Professor of Neurology at the Mayo Clinic College of Medicine, cites different size thresholds, a more aggressive attitude towards intervention and an emphasis on patient perspective [70]. He recommends that treatment be considered for UIAs >12 mm unless the patient is older or has significant comorbidities, for UIAs 7–12 mm, in particular those that are symptomatic, in specific locations or are harbored among younger patients, and for UIAs <7 mm on a case-by-case basis especially for younger patients.

Further, while some surgeons recommend microsurgical clipping as the first treatment choice because of its proven long-term efficacy [69], the prevailing thought seems to favor endovascular coiling as the more amenable first-line treatment due to its low morbidity, mortality, and complication rates [71]. Still, this dispute overshadows the fact that each technique has its own strengths and weaknesses and that these treatment decisions should be evaluated individually based on patient and aneurysm characteristics.  Table 2 summarizes the treatment recommendations referenced above.

## 4. Conclusion

 Management of UIAs is characterized by an outwardly simple comparison between risk of observation and risk of intervention. However, deeper analysis shows that these situations are anything but simple. In this paper, we have summarized the patient-specific and aneurysm-specific factors that affect the natural history of these lesions. Additionally, safety and efficacy data are presented for both surgical clipping and endovascular coiling to illustrate the risks and benefits of treatment. Review of current treatment guidelines can provide practitioners with a framework to make appropriate treatment decisions when faced with UIA cases, but each patient should be approached on a case-by-case basis so as to consider the multifactorial nature of the disease.

## Figures and Tables

**Table 1:** Key findings related to the natural history data.

Risk factor	Key findings	References
Size	(i) Larger UIAs have greater RR (ii) Aneurysm size is a significant independent predictor of RR (iii) Defining a critical size threshold for aneurysm rupture remains difficult	ISUIA investigators 1998 [7] Wiebers et al. 2003 [8] Ishibashi et al. 2009 [9]

Enlargement	(i) In most cases, IAs are larger at time of rupture than at initial diagnosis (ii) Larger UIAs are more likely to grow (iii) Larger UIAs → greater growth risk → increased RR	Yasui et al. 1996 [10] Burns et al. 2009 [1] Matsubara et al. 2004 [11]

Previous SAH	(i) Prior history of aneurysmal SAH increases future RR (ii) Aneurysms <7 mm have an increased RR with prior history of SAH	ISUIA investigators 1998 [7] Wiebers et al. 2003 [8]

Location	(i) Posterior circulation aneurysms are widely considered to be more hazardous (ii) Include basilar artery, posterior cerebral artery, and vertebrobasilar distribution (iii) Intracavernous IAs are more benign	Weir et al. 2002 [12] Wermer et al. 2007 [13] Kupersmith et al. 1992 [14]

Morphology	(i) Multiple lobulations or loculations increases RR (ii) High dome : neck ratio increases RR (iii) Aneurysm angle, undulation index, and nonsphericity index are all predictors of aneurysm rupture	Hademenos et al. 1998 [15] Beck et al. 2003 [16] Dhar et al. 2008 [17] Raghavan et al. 2005 [18]

Patient characteristics	(i) Age, sex and comorbidities will influence aneurysmal RR (ii) Female sex and cigarette smoking are independent predictors of both UIA formation and growth (iii) These factors are especially important when making decision on whether to treat	Nahed et al. 2005 [19] Juvela et al. 2001 [20]

**Table 2:** Treatment recommendations.

Source	Recommendations
American Heart Association's Stroke Council	(1) Treatment of small intracavernous ICAs is not advised. Large intracavernous ICAs should be considered, taking into account age and symptoms. (2) Symptomatic intradural ICAs should be considered for treatment. However, large or giant symptomatic aneurysms require exhaustive consideration of individual patient characteristics and the expertise of surgeon and facility. (3) Patient with prior history of aneurysmal SAH should be considered for treatment, especially for UIAs at the basilar apex (4) Asymptomatic aneurysms <10 mm in patients without prior history of aneurysmal SAH generally should be observed. Special consideration should be given to patient with family history or unique hemodynamic factors. (5) Asymptomatic UIAs ≥10 mm warrant strong consideration for treatment.

Mayo Clinic College of Medicine	(1) With rare exception, all symptomatic UIAs should be treated. (2) Small incidental aneurysms <5 mm should be managed conservatively. (3) UIAs >5 mm in patients younger than 60 years of age should be strongly considered for treatment. (4) UIAs >10 mm in patients younger than 70 years of age should be strongly considered for treatment. (5)Microsurgical clipping rather than endovascular coiling should be the first-choice treatment in low-risk cases.

Columbia University	(1) Patients under the age of 45 should be strongly considered for treatment with exceptions being small, anterior circulation UIAs. (2) Aneurysms >12 mm should be strongly considered for treatment, except in older patients and those at high risk of intervention (3) Conservative management is recommended for patients with UIAs <5 mm in anterior circulation and <3 mm in posterior circulation, except if patient is below the age of 45 or has strong family history (4) Factors to be considered in all UIA cases include aneurysmal factors such as daughter sac formation or multiple lobes and patient factors such as family history, age, and comorbidities (5) Antihypertensive and smoking cessation therapies should be recommended to all patients with UIAs (6) Follow-up, noninvasive imaging with MRA or CTA should be done at 6 and 12 months after diagnosis, followed by yearly imaging for at least 3 years to monitor enlargement.
